# Low-Light Anoxygenic Photosynthesis and Fe-S-Biogeochemistry in a Microbial Mat

**DOI:** 10.3389/fmicb.2018.00858

**Published:** 2018-04-27

**Authors:** Sebastian Haas, Dirk de Beer, Judith M. Klatt, Artur Fink, Rebecca McCauley Rench, Trinity L. Hamilton, Volker Meyer, Brian Kakuk, Jennifer L. Macalady

**Affiliations:** ^1^Max Planck Institute for Marine Microbiology, Bremen, Germany; ^2^Department of Oceanography, Dalhousie University, Halifax, NS, Canada; ^3^Department of Earth and Environmental Sciences, University of Michigan, Ann Arbor, MI, United States; ^4^Geosciences Department, Pennsylvania State University, University Park, PA, United States; ^5^Department of Plant and Microbial Biology, University of Minnesota, Minneapolis, MN, United States; ^6^Bahamas Caves Research Foundation, Marsh Harbour, Bahamas

**Keywords:** anoxygenic photosynthesis, green sulfur bacteria, low-light photosynthesis, sulfide scavenging, microbial mat, bacteriochlorophyll *e*, iron-sulfur-cycling, Proterozoic ocean

## Abstract

We report extremely low-light-adapted anoxygenic photosynthesis in a thick microbial mat in Magical Blue Hole, Abaco Island, The Bahamas. Sulfur cycling was reduced by iron oxides and organic carbon limitation. The mat grows below the halocline/oxycline at 30 m depth on the walls of the flooded sinkhole. *In situ* irradiance at the mat surface on a sunny December day was between 0.021 and 0.084 μmol photons m^-2^ s^-1^, and UV light (<400 nm) was the most abundant part of the spectrum followed by green wavelengths (475–530 nm). We measured a light-dependent carbon uptake rate of 14.5 nmol C cm^-2^ d^-1^. A 16S rRNA clone library of the green surface mat layer was dominated (74%) by a cluster (>97% sequence identity) of clones affiliated with *Prosthecochloris*, a genus within the green sulfur bacteria (GSB), which are obligate anoxygenic phototrophs. Typical photopigments of brown-colored GSB, bacteriochlorophyll *e* and (β-)isorenieratene, were abundant in mat samples and their absorption properties are well-adapted to harvest light in the available green and possibly even UV-A spectra. Sulfide from the water column (3–6 μmol L^-1^) was the main source of sulfide to the mat as sulfate reduction rates in the mats were very low (undetectable-99.2 nmol cm^-3^ d^-1^). The anoxic water column was oligotrophic and low in dissolved organic carbon (175–228 μmol L^-1^). High concentrations of pyrite (FeS_2_; 1–47 μmol cm^-3^) together with low microbial process rates (sulfate reduction, CO_2_ fixation) indicate that the mats function as net sulfide sinks mainly by abiotic processes. We suggest that abundant Fe(III) (4.3–22.2 μmol cm^-3^) is the major source of oxidizing power in the mat, and that abiotic Fe-S-reactions play the main role in pyrite formation. Limitation of sulfate reduction by low organic carbon availability along with the presence of abundant sulfide-scavenging iron oxides considerably slowed down sulfur cycling in these mats.

## Introduction

A significant part of the Earth’s biosphere is exposed to regimes of extreme energy limitation, such as the terrestrial deep biosphere, the deep sea, and deep zones of most marine sediments. Energy limitation may have been even more significant on early Earth in the absence of oxygenic photosynthesis and the associated production of electron acceptors such as molecular oxygen and oxidized forms of nitrogen, iron, and sulfur. Although studies of low-energy communities are technically challenging and therefore relatively rare, they demonstrate how biotic and abiotic processes compete and interact, and may approximate conditions on early Earth and other planetary bodies.

The lower limits of biotic utilization of light are reached by anoxygenic photosynthesis, especially by members of the monophyletic clade of the GSB (*Chlorobiaceae*), which are obligate anaerobes and obligate anoxygenic phototrophs. They can proliferate at the interface of the sulfidic and photic zone in meromictic or eutrophic lakes (Montesinos et al., 1983; Mori et al., 2013; Crowe et al., 2014) or in microbial mats (Engel et al., 2004; Lau et al., 2009). The extremely low-light-adapted strain *Prosthecochloris phaeobacteroides* BS1 (formerly *Chlorobium phaeobacteroides* BS1; Manske et al., 2005; Imhoff and Thiel, 2010; Marschall et al., 2010) forms monospecific assemblages in the Black Sea chemocline and has been shown to fix inorganic carbon by the phototrophic oxidation of sulfide at light intensities as low as 0.015 μmol photons m^-2^ s^-1^, which is five orders of magnitude lower than daylight (Manske et al., 2005). With estimated *in situ* carbon fixation rates as low as 200 to 1800 ng C m^-2^ d^-1^, however, they have virtually no quantitative effect on the carbon and sulfur cycles in their habitat (Manske et al., 2005). Low-light-adapted GSB are referred to as brown-colored GSB, a non-monophyletic group of low-light specialists (Montesinos et al., 1983) that possess the BChl *a* and *e* as well as the carotenes isorenieratene and β-isorenieratene (Glaeser et al., 2002). BChl *e* and these carotenes are responsible for light-harvesting and energy transfer to BChl *a* in model GSB strains (Overmann et al., 1992; Hauska et al., 2001). GSB with this combination of pigments have an *in vivo* absorption maximum at 505 nm (Overmann et al., 1992). Since the physical light attenuation of water is lowest between 400 and 500 nm (Sogandares and Fry, 1997), they are able to thrive at great water depths.

Anoxygenic phototrophs can use a variety of electron donors, including nitrite (Griffin et al., 2007), ferrous iron (Widdel et al., 1993; in GSB: Heising et al., 1999), molecular hydrogen or reduced sulfur compounds (Pfennig, 1975; Overmann and Pfennig, 1989). Hydrogen sulfide is the electron donor most commonly used by GSB. It is converted according to the following stoichiometry:

(1) $$2{\mathrm{HS}}^{-}+{\mathrm{CO}}_{2}+2H^{+}\to 2S^{0}+{\mathrm{CH}}_{2}O+H_{2}O$$

Hydrogen sulfide is produced biologically by sulfate reduction, a process most prominent in marine sediments, but also frequently observed in biofilms and microbial mats (Canfield and Des Marais, 1991; Kühl and Jørgensen, 1992; Visscher et al., 1992). Wieland et al. (2005) demonstrated how photosynthates in a microbial mat induced significantly increased sulfate reduction rates (SRRs) during the day. CO_2_ produced by sulfate reduction in turn enhanced photosynthesis. The same study also showed how large amounts of Fe(III) can affect sulfur cycling by precipitating sulfide. In the present study, we describe how a similar effect can slow down sulfur cycling in a mat that is significantly more limited in light and organic carbon.

Green sulfur bacteria may produce sulfate from hydrogen sulfide or more oxidized sulfur compounds (Overmann and Pfennig, 1989), but can also produce elemental sulfur (S^0^), which is deposited extracellularly (Pfennig, 1975). The production of sulfur compounds of intermediate oxidation states rather than sulfate is common also to non-phototrophic sulfide oxidation processes. In fact, the presence of incompletely oxidized sulfur in the form of CRS can be used as an indication for sulfide oxidation (e.g., Thode-Andersen and Jørgensen, 1989; Holmkvist et al., 2011; Lichtschlag et al., 2013), because sulfate reduction typically does not produce sulfur compounds of intermediate oxidation states. In the absence of light, biological sulfide oxidation typically requires either oxygen, nitrate or nitrite as electron acceptor.

Blue holes are sinkholes: vertical, water-filled karst features open to the surface (Mylroie et al., 1995). Inland blue holes of the Bahamas are anchialine caves (Iliffe, 2000), landlocked bodies of water with subterranean connections to the ocean containing meromictic water columns with an upper freshwater lens separated from a lower saltwater column by a stable halocline (Seymour et al., 2007; Gonzalez et al., 2011). Each blue hole displays distinct geochemical traits, making them unique natural laboratories that allow exploring the limits of photosynthesis and the interplay between biotic and abiotic sulfur cycling. Inland blue holes are therefore of particular interest for geomicrobiology, because the unique geochemical features of each blue hole (e.g., depth of halocline, concentration of sulfide, water flow rates, organic matter input) are associated with the formation of specific types of microbial communities (Gonzalez et al., 2011). Few studies have been conducted on the microbiology of inland blue holes to date (Bottrell et al., 1991; Schwabe and Herbert, 2004; Macalady et al., 2010; Gonzalez et al., 2011).

A thick, orange-colored microbial mat with a thin green top layer was discovered in the suboxic, sulfidic water below the halo-chemocline of MBH, The Bahamas (Gonzalez et al., 2011). The green color of surface cells and an abundance of GSB-affiliated 16S rRNA genes in clone libraries suggested the mats were phototrophic (Gonzalez et al., 2011). *δ-Proteobacteria* were also highly abundant in the clone libraries, suggesting that sulfate reduction might be an important process in the mats (Gonzalez et al., 2011). We aimed to test the hypothesis that the mats are phototrophic and dominated by GSB that are optimally adapted to the quality and low intensity of the ambient light. Secondly, we aimed to test whether the sulfide needed for the GSB activity is supplied by sulfate reduction in the mats. We used a highly sensitive light detection system to quantify ambient irradiance and determine the light quality. The ambient spectra were compared with the absorption spectra of photopigments from the mats. Activities were determined by light dependency of carbon uptake and sulfate reduction rate measurements. To constrain the Fe-S-cycling in the mats, solid phase fractions of the iron and sulfur pools were quantified.

## Materials and Methods

### Study Site and Mat

**FIGURE 1:**
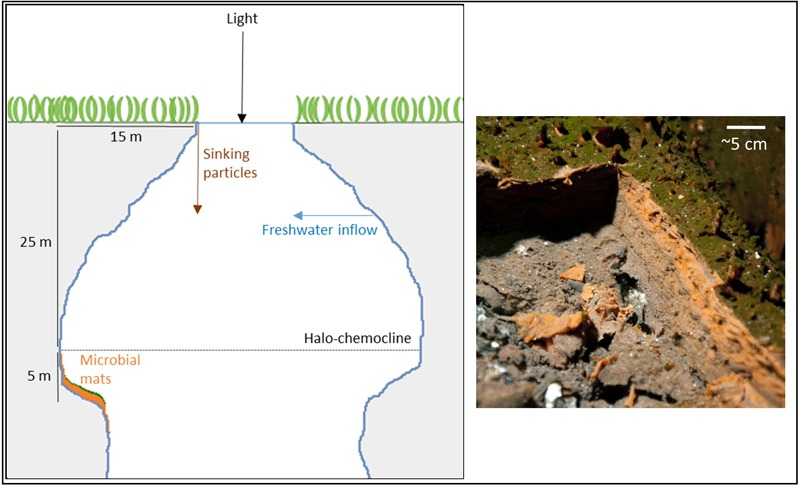
Schematic drawing of Magical Blue Hole and a picture of the microbial mat of which a piece was cut out to reveal its three-layered structure.

Magical Blue Hole (also known as Cherokee Road Extension Blue Hole, 26°22′31.96″N, 77°6′14.91″W) is an anchialine sinkhole on Abaco Island (The Bahamas). It is approximately 80 m deep and has a surface diameter of approximately 10 m. A stable halo- and chemocline is present at 25 m depth, separating oxic freshwater from anoxic, sulfidic seawater (Gonzalez et al., 2011). Below the surface, the diameter widens and the cave is roughly 40 m wide at 30 m depth. The narrow entry and the shading effect of the overhanging cave walls result in low light intensities below the halo-chemocline. The cave walls below the halo-chemocline are extensively covered by a thick microbial mat. It grows thickest (up to ∼6 cm) on a ledge in the otherwise vertical cave wall situated at approximately 30 m depth. The mat is stratified with a thin green layer of fluffy biological material on top (∼1 mm), a gelatinous orange middle layer (up to ∼3 cm) and a brown bottom-layer (up to ∼3 cm; **Figure 1**).

### Sampling Techniques

Geochemical parameters of the water column were measured in vertical profiles. Water samples were collected in lockable plastic syringes by a diver and sterile-filtered (0.2 μm) immediately after sampling. Samples for dissolved inorganic and organic carbon analysis were stored at 4°C while samples for ammonium (NH_4_^+^) and nitrate (NO_3_^-^) quantification (*n* = 5 for 30 m and 40 m; *n* = 2 for remaining depths) were stored frozen. Total sulfide (H_2_S + HS^-^ + S^2-^) and sulfate (SO_4_^2-^; *n* = 3 for all depths, respectively) samples were preserved by mixing 1:1 (v/v) with 20% Zn-acetate solution.

Samples for incubation with ^13^C-labeled substrates and sub-samples for photosynthetic pigment analysis (*n* = 3) were taken by vacuuming the upper green part of the mat into lockable syringes. For clone library construction, a large piece of mat was cut out with a knife and immediately placed into a light-shielded and airtight plastic box by a diver for transport to the laboratory. Clone library samples were separated into the three natural layers based on location and color, preserved with RNAlater and stored at 4°C until analysis.

Microbial mat samples from around 30 m water depth were taken with push-cores, which were immediately closed by a diver with airtight plugs. Push-cores used to sample for pigment analysis (*n* = 3; core lengths: 1.2–5.7 cm) and ^35^S incubation experiments (*n* = 10; lengths: 3.6–5.4 cm) were wrapped in black tape to shield the samples from light. Cores for solid phase analyses were immediately cut into 0.6 cm slices (AVS/ CRS/ sulfate; lengths: 3.6–5.4 cm) and 0.25 cm slices (dithionite reactive iron/ S^0^; lengths: 3.5–4.5 cm) and fixed in 4 mL 5% Zn-acetate and 3 mL 20% Zn-acetate, respectively. Along with samples for pigment analysis, they were stored frozen. Samples for porosity (*n* = 4) and density (*n* = 1) measurements were also taken with push-cores.

### Solid Phase Analyses

Mat porosity was determined as weight reduction after drying at 60°C in four mat cores (result: 0.92 ± 0.02). Density was determined as wet weight per mat volume. The density average of 0.6 cm/ 1 mL slices (*n* = 8) from one core sample (1.11 ± 0.12 g cm^-3^) was used for conversions of mat weight into volume.

Acid volatile sulfide (AVS = FeS + Fe_3_S_4_ + H_2_S: Cornwell and Morse, 1987) and CRS (CRS = S^0^ + FeS_2_: Cornwell and Morse, 1987) were extracted from six mat core samples in a two-step HCl and Cr(II) distillation according to Fossing and Jørgensen (1989). After distillation of the pellets, AVS and CRS trapped as ZnS were determined according to Cline (1969). Porewater sulfate content was quantified in the supernatant after a centrifugation step, which preceded these distillations. The detection limit was at 12.9 nmol S cm^-3^. From five parallel cores, S^0^ was extracted by methanol and measured as cyclo-S_8_ by UPLC (Waters, United States) as described previously (Zopfi et al., 2004). Pyrite-S was defined as the difference between CRS and S^0^.

A citrate-acetate-dithionite solution was used to extract iron from three mat samples. The procedure extracts most crystalline and amorphous Fe(III) as well as FeS (Kostka and Luther, 1994). Extraction was preceded by a centrifugation step after which the supernatant was discarded. Since Zn-acetate was used to preserve the samples, iron concentrations may be underestimated due to possible formation of non-extractable Fe-acetate. Dithionite extracts were analyzed with the ferrozine method according to Viollier et al. (2000).

### Geochemical Water Analyses

Standard procedures were used to analyze chemical compounds in samples from the water column and in microbial mat extracts. Total sulfide was measured according to Cline (1969). Sulfate ion concentration was analyzed with ion chromatography (761 Compact Ion Chromatographer including 812 valve unit and 838 Advanced Sample Processor, Metrohm, Germany). Phosphate, nitrate and ammonium were quantified with a continuous flow analyzer (San^++^, Skalar, Netherlands) using standard detection methods (Hansen and Koroleff, 2009). DIC was calculated as the difference between total carbon and non-purgeable organic carbon measured using a Total Carbon Analyzer (TOC-5000A, Shimadzu, Japan) after acidification and N_2_-purging.

### Photosynthetic Pigment Analysis

Frozen mat cores were horizontally sliced with a resolution of 3 mm. Pigments from both core slices and vacuumed surface layer samples were extracted with acetone as described previously (Al-Najjar et al., 2012). The filtered extract containing the pigments was then injected into a reversed-phase High-performance liquid chromatograph (HPLC; 2695 Separations Module, Waters, United States). The pigments were separated according to Wright (1991) and absorption spectra were measured using a 996 Photodiode Array Detector. Identification of pigments was based on RT and absorption spectra. A *β*-carotene standard (beta-122; 0.813 mg L^-1^ in 100% acetone, Sigma-Aldrich, United States) and freeze-dried pure culture material of a low-light-adapted *Prosthecochloris phaeobacteroides* BS1strain isolated from the Black Sea chemocline by Overmann et al. (1992) (DSMZ Braunschweig) were analyzed in parallel as standards.

### Isotope Incubations for Process Rate Determination

Rates of sulfate reduction, photoautotrophic carbon uptake and photoheterotrophic carbon uptake were assessed by incubations with isotopic labels. To assess SRRs inside the mat, the whole-core injection method based on radioactively labeled sulfate was carried out (Jørgensen, 1978). Under N_2_-atmosphere, 4 μl per cm^3^ sample of radioactively labeled ^35^SO_4_^2-^ in Na_2_SO_4_ carrier solution (∼33 kBq cm^-3^) were vertically injected into push-cores (20 mm diameter) containing up to 20 mL freshly sampled microbial mat. Incubation under anoxic conditions and approximate *in situ* temperature was stopped after four (*n* = 6) and six (*n* = 4) hours by horizontally slicing the cores into 6 mm pieces and immediately mixing them 1:1 (v/v) with 20% Zn-acetate to stop sulfate reduction and fix hydrogen sulfide as ZnS. After cold Cr(II) distillation (Kallmeyer et al., 2004; as modified by Røy et al., 2014), SRRs were determined (Jørgensen, 1978). A detection limit of 43.73 radioactive decays per minute (95% confidence interval) was calculated based on means and standard deviations of sample blanks prepared (Kallmeyer et al., 2004).

**FIGURE 2:**
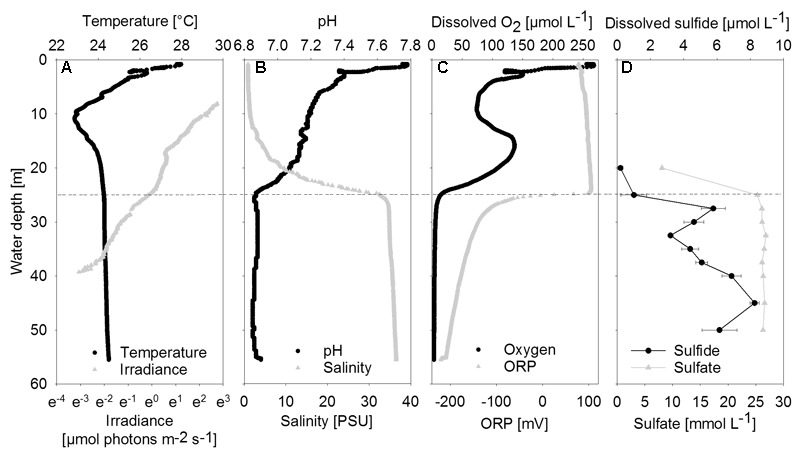
Vertical profiles of chemical and physical parameters in the center of the water column. **(A)** Temperature and irradiance. Irradiance is plotted on a logarithmic scale. **(B)** PH and Salinity. **(C)** Dissolved oxygen and ORP, oxidation-reduction potential. **(D)** Dissolved sulfide and sulfate shown as mean concentrations (*n* = 3); error bars represent the standard deviation. The halo-chemocline is indicated by the dashed line at 25 m.

To determine phototrophic carbon uptake, parts of the green surface layer of the mat were incubated *in situ* in cave water amended with labeled ^13^C-DIC (NaH^13^CO_3_) or ^13^C-acetate (^13^CH_3_^12^CO_2_Na) at 30 m depth in the center of the cave (maximum irradiance: 0.27 μmol photons m^-2^ s^-1^; **Figure 2A**) for different time periods. Under anoxic N_2_-atmosphere and protected from high light exposure, samples were suspended in cave water (collected oxygen-free next to the mat) to a final volume of 500 ml. To ensure that the medium was completely anoxic, 18 μl of 1 M Na_2_S were added to a final sulfide concentration of 84 μmol L^-1^ at pH 6.85 ± 0.35. Glass vials (6 mL) with plastic lids featuring integrated septa were filled with medium containing the inoculum and isotope label depending on the treatment. Vials were supplemented with 73.8 nmol of ^13^C-labeled DIC or ^13^C-labeled acetate, which resulted in ^13^C-labeling percentages of 10.5% for DIC and 50% for acetate, calculated based on observed *in situ* DIC concentrations and by assuming negligible *in situ* amounts of acetate. Triplicate aliquots for determination of natural isotope abundance were preserved before label addition. The ^13^C content of three incubation vials was immediately preserved after addition of label (*t* = 0). The remainder of the closed incubation vials were incubated in the center of the blue hole at 30 m depth. Dark controls were incubated in light-shielded vials. One set of vials (low-light incubated: *n* = 3; dark controls: *n* = 3) was recovered after approximately 2 days and a second set after 6 days. At the prevailing daylight length of 10.5 h, this corresponded to an incubation time in natural light of 24 h and 60 h, respectively. All incubations were terminated by filtration through pre-combusted (3 h, 450°C) GF/F-filters (0.7 μm pore size), which were then dried at 60°C and subsequently treated and analyzed as described in Halm et al. (2012). Rates of total DIC and acetate assimilation were calculated as the increase in ^13^C/^12^C over time, corrected for natural abundance and ^13^C-labeling percentage.

Carbon uptake rates per single cell and per mat area were calculated assuming that all cells were phototrophic and covering the mat surface without gaps. Phototrophic cell numbers per incubation vial were estimated based on total carbon determined by isotope ratio mass spectrometry per incubation vial and using literature values of cell radius (0.6125 μm in Black Sea strain *P. phaeobacteroides* BS1 cells cultivated at 0.1 μmol photons m^-2^ s^-1^; Manske et al., 2005) and carbon content (106 fg μm^-3^; Nagata, 1986). They were corrected for carbon content determined in *in situ* cave water in the same way. To calculate quantum yields of photosynthesis - the amount of carbon fixed per incoming photons - we assumed a total absorptive cell surface of 0.716 cm^2^ per incubation vial. This was based on the product of the mean phototrophic cell numbers per vial (1.16 × 10^8^ cells vial^-1^) and an assumed single-cell absorption cross section (= average light absorbing cell surface) of 0.62 μm^2^ (Black Sea *P. phaeobacteroides*: Marschall et al., 2010).

### Phylogenetic Analyses

Mat microbial DNA was extracted from mat samples using chloroform-phenol extraction and 16S rRNA genes were amplified by PCR using Bacteria-specific 27f and 1492r primers as described in Macalady et al. (2008). One library was constructed for each mat layer. Potential chimeras were excluded from further analysis. Full length and partial sequences for the green layer (*n* = 74), orange layer (*n* = 65) and brown layer (*n* = 67) were obtained from the respective libraries. Sequences were aligned with the Silva aligner available at http://www.arb-silva.de, imported into ARB (Ludwig et al., 2004) and manually refined. Operational taxonomic units (OTUs) were identified using a sequence identity threshold of 97% (0.03) using mothur (v.1.39.5) (Schloss et al., 2009). Taxonomy was assigned using BlastN (Altschul et al., 1997) and ARB (Ludwig et al., 2004). For phylogenetic analyses of sequences affiliated with *Chlorobi* and δ*-Proteobacteria*, the top BLAST matches and nearest relative to each OTU in the ARB database were included. Representative sequences were added to an existing 16S rRNA alignment in ARB, and manually refined. Maximum likelihood analyses were performed using RAxML (Stamatakis et al., 2008) with 1000 bootstrap replicates and the general time-reversible model with G+I rate variation as determined by JModelTest v.2.1.10 (Darriba et al., 2012). The resulting trees were viewed and edited using iTOL^1^ (Letunic and Bork, 2016). The 16S rRNA gene sequences recovered in this study were submitted to the GenBank database and assigned the following accession numbers: MG601241–MG601445.

### *In Situ* Measurements of Physico-Chemical Parameters

For measurements of physico-chemical parameters, a data-logging device was designed and constructed to measure (i) downwelling scalar irradiance and (ii) physico-chemical parameters as well as (iii) spectra of downwelling light available to the mat. Irradiance was measured with a modified DOMS (Weber et al., 2007). A scalar irradiance sensor (21.21 mm^2^ sensing surface; US-SQS/LI, Walz, Germany) with a glass fiber was attached to a H5702-50 PMT (Hamamatsu Photonics, Japan). The control voltage and thereby the PMT sensitivity was scaled to ambient light conditions by a diver during measurements using a magnetic switch. The scalar irradiance sensor installed on the PMT measured irradiance over an angle of approximately 270°.

The DOMS logger, its NiMH rechargeable battery, the modified PMT, a cosine-corrected PAR sensor (QCP-2000; Biospherical Instruments, United States) and a multiparameter sonde (YSI 5200A, YSI, United States) equipped with sensors for pressure, ORP, pH, dissolved oxygen, temperature and salinity, were mounted on a vertical frame. This array could then be lowered into the blue hole with a rope for vertical profiling (descent rate: 4.3 m min^-1^) or made neutrally buoyant and moored to the cave wall. By attaching both PAR- and PMT-sensors in one plane and upward orientation, parallel signals of the two sensors were obtained during the descent of the array through upper cave regions, where sufficient irradiance covered the sensitivity ranges of both light sensors. Using the PAR sensor’s internal calibration, these overlapping data could be used to create a calibration curve for PMT measurements at greater depths.

The spectral quality of the light reaching the mat was determined by using a filter wheel containing optical longpass filters (FSR-GG-400 nm and -475 nm, FSR-OG-530 nm and -590 nm, FSR-RG-645 nm; Newport, United States) in front of the sensor. The sensor was shaded from below with a movable cup and the filter wheel was operated manually by a diver. Based on the amplitude of irradiance reduction measured by the sensor after each filter switch, an *in situ* wavelength spectrum was obtained. The filters reduced the light intensity above their respective threshold by 10% (manufacturer information), for which the spectrum was corrected. The PMT covered an irradiance spectrum of 200–700 nm with sensitivity dropping above and below this spectrum. Within this spectrum, sensitivity was approximately even, except for slightly increased sensitivity in the wavelength range 500–680 nm (manufacturer information) that may have led to a small overestimation (<2%) of the percentage of light from this wavelength range.

## Results

### Water Column Geochemistry and Irradiance

Between 20 and 25 m depth, a distinct halo-chemocline was observed, below which the water was sulfidic and anoxic (**Figure 2**). Dissolved oxygen and temperature profiles displayed negative anomalies at about 10 m depth, probably due to lateral water inflow. Below the halo-chemocline, most chemical parameters and temperature remained approximately constant. Deviating from this trend, sulfide displayed two maxima, namely at 27.5 m, i.e., directly below the halo-chemocline and close to the depth of highest mat abundance, and at 45 m. There was no detectable sulfide above the halo-chemocline. Close to the depth of thickest mat growth, we observed a minimum in sulfide concentration (**Figure 2D**).

Nutrient and dissolved organic carbon concentrations were low (data not shown). Phosphate concentrations ranged between 0.2 μmol L^-1^ in the deep water (40 m) and 0.5 μmol L^-1^ in the halo-chemocline (25 m). Dissolved inorganic nitrogen was present below the halo-chemocline in the form of ammonium (approximately 10 μmol L^-1^) and above the halo-chemocline as nitrate (3 μmol L^-1^). Dissolved non-purgeable organic carbon concentrations ranged from 171 μmol L^-1^ in the deep water (47 m) to 258 μmol L^-1^ in the oxic water column (9 m) with intermediate concentrations around 30 m.

**FIGURE 3:**
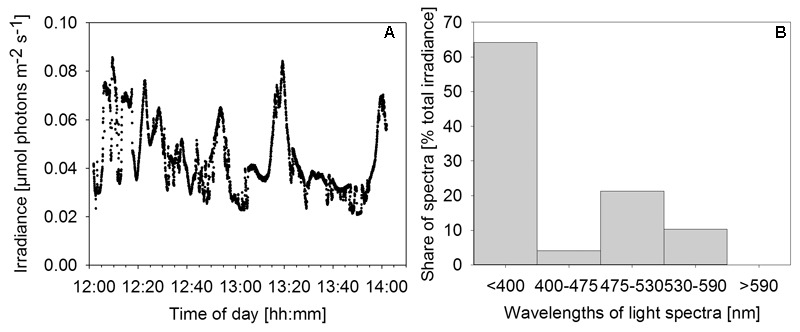
Irradiance and relative wavelength abundance *in situ*. **(A)** Time series of *in situ* irradiance (270°) available to the mat over 2 h starting at noon on December 1st, 2013. Weather: sunny with occasional clouds. **(B)** Percentage share of wavelength ranges in total downwelling light reaching the mat as determined by application of a series of longpass filters.

Downwelling scalar irradiance declined with depth above 17 m and slightly more steeply with depth between 17 and 25 m (**Figure 2A**). Below the halo-chemocline, the light attenuation just below 25 m depth was stronger compared to the zone around 30 m depth. Irradiance at 30 m depth in the cave center was 0.27 μmol photons m^-2^ s^-1^. At the same depth near the cave wall - the site of thickest mat growth - irradiance was notably lower (**Figure 3A**), in the range of 0.021 to 0.084 μmol photons m^-2^ s^-1^ during 2 h of measurement on a sunny day around noon. Occasional clouds caused sudden decreases in irradiance.

Measurements with a series of longpass filters revealed that the light attenuation varied substantially with wavelength. Intriguingly, 64% of the total irradiance available to the mat was light in the UV region of the spectrum (<400 nm). A second peak of relative wavelength abundance (21%) was in the green part of the spectrum (475–530 nm). Red/near-IR light (>590 nm) did not reach the mat surface (**Figure 3B**).

### Clone Libraries Dominated by Green Sulfur Bacteria and δ-*Proteobacteria*

**FIGURE 4:**
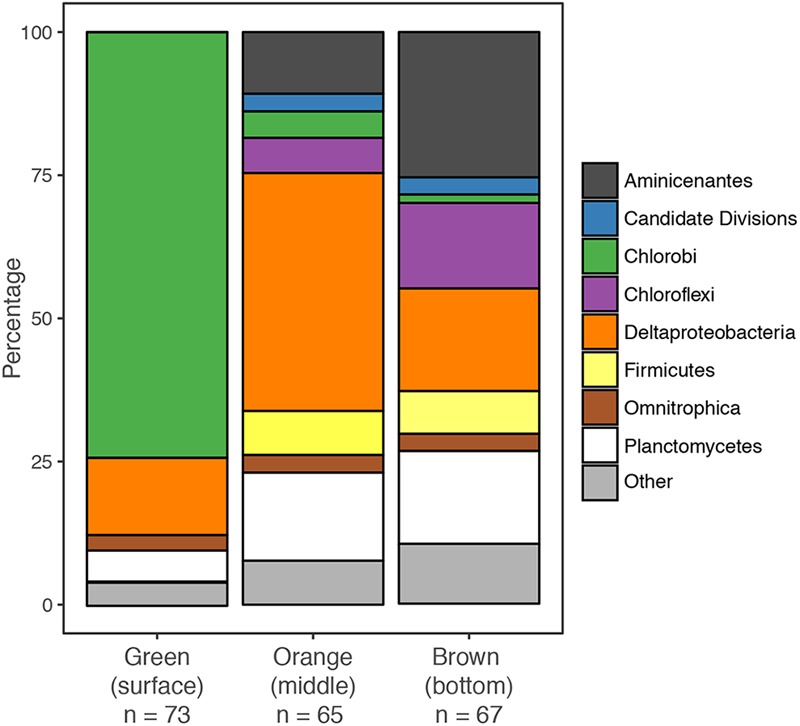
Community structure of individual mat layers based on bacterial 16S rRNA clone libraries.

We created separate 16S rRNA clone libraries from the green, the orange and the brown mat layers (**Figure 4**). The vast majority of the clones (74%) from the thin green upper layer were affiliated with GSB (*Chlorobi*). GSB were much less abundant in the middle, orange layer, which was dominated by δ*-Proteobacteria* related clones (37%). In the brown bottom layer, clones affiliated with *Aminicenantes* (OP8), δ*-Proteobacteria, Planctomycetes* and *Chloroflexi* were found most abundant, while GSB (1%) were virtually absent.

The GSB-affiliated 16S rRNA gene sequences shared >97% sequence identity and clustered together in the phylogenetic tree (Supplementary Figure S1). The MBH GSB cluster was situated in a wider cluster of *Prosthecochloris* reference sequences and the closest relative we identified was a clone from the chemocline of Sawmill Sink, a blue hole on the same island as MBH (Macalady et al., unpublished data). Unlike GSB, most δ*-Proteobacteria* clones did not cluster with each other, but were each affiliated with a variety of uncultured reference sequences from diverse habitats (Supplementary Figure S2). A majority of clones were widely clustered with *Desulfobacteraceae*, and a few more with *Syntrophobacteraceae*.

### The Mat Contains the Photopigments Bacteriochlorophyll *e* and (*β*-)isorenieratene

**FIGURE 5:**
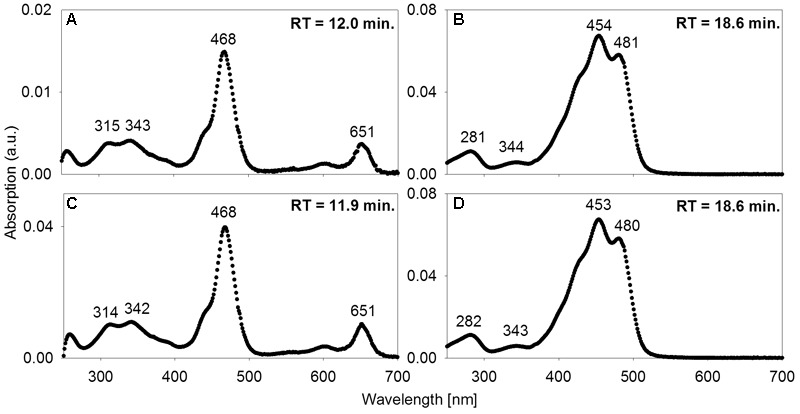
Absorption spectra of acetone-extracted photosynthetic pigments. **(A,B)** from the mats; **(C,D)** and from freeze-dried culture material of low-light-adapted *Prosthecochloris phaeobacteroides* BS1 strain from the Black Sea. Wavelengths [nm] of absorption maxima are indicated as well as RT in the HPLC column.

High-performance liquid chromatography analysis was used to assess whether the mat contains photopigments with absorption properties congruent with the *in situ* light quality. To a depth of 57 mm below the mat surface, a carotene identified as either isorenieratene or β*-*isorenieratene was abundant. It was detected in both *P. phaeobacteroides* BS1 pure culture reference material and in the mat based on identical absorption spectra and RTs (**Figures 5B,D**). Based on difference in RT, it was clearly distinguished from β-carotene, which has the same absorption spectrum as β-isorenieratene and isorenieratene (Takaichi, 2000; Fuciman et al., 2010). Further distinction could not be made, because the reference culture contained both β-isorenieratene and isorenieratene.

Additionally, we found BChl *e* to be abundant in the upper 0.8 to 15 mm of the three core samples as well as in most samples from vacuum-collected green mat surface material. It was identified based on identical absorption spectra and RT of a pigment from *P. phaeobacteroides* BS1 biomass. The absorption spectrum matched published *ex vivo* spectra of BChl *e* (Gloe et al., 1975; Borrego et al., 1999), displaying a maximum at 468 nm, a smaller peak at 651 nm and a double peak at 315/343 nm (**Figures 5A,C**). In both *P. phaeobacteroides* BS1 and mat samples, we detected a variety of BChl *e* homologs (Borrego and Garcia-Gil, 1994) that all displayed the described absorption spectrum, but had different RTs ranging from 11.2 to 16.9 min. Besides BChl *e* we did not detect any type of BChl or chlorophyll.

### Low-Light Dependent Uptake of ^13^C-Labeled DIC and Acetate

**FIGURE 6:**
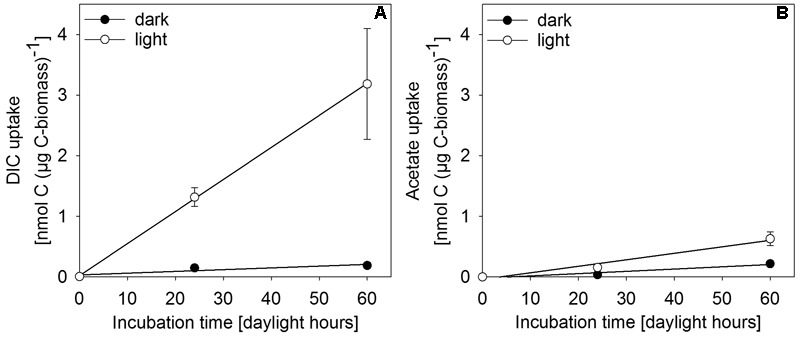
Incorporation of **(A)** DIC and **(B)** acetate as uptake per carbon biomass of upper-layer mat material during incubation in *in situ* water at 30 m depth in the center of MBH. Dark controls were shielded from light during incubation. Error bars indicate standard deviation of the mean of parallel incubations (*n* = 3).

*In situ* incubation with ^13^C-labeled DIC or acetate revealed light-dependent uptake of inorganic and organic carbon (at 0.27 μmol photons m^-2^ s^-1^) by surface-layer mat bacteria. Light dependent uptake of DIC was sevenfold higher than uptake of acetate (**Figure 6**). Dark uptake rates of both acetate and DIC were considerably lower.

Single-cell net phototrophic uptake rates were determined from estimated cell numbers per incubation vial and experimentally determined carbon uptake rates (DIC: 7.1 × 10^-9^ nmol C cell^-1^ h^-1^; acetate: 1.2 × 10^-10^ nmol C cell^-1^ h^-1^). Assuming that the mat surface is homogeneously covered by active photoautotrophic cells, we used single-cell rates to estimate an *in situ* autotrophic carbon fixation rate of 14.5 nmol C cm^-2^ d^-1^. If photosynthetic sulfide oxidation strictly proceeded according to Eq. 1, this corresponded to a phototrophic sulfide oxidation rate of 29.0 nmol S cm^-2^ d^-1^. The photosynthetic quantum yield under experimental conditions was calculated from the photon flux of light between 475–530 nm (1.93 × 10^-5^ μmol photons m^-2^ s^-1^) per total incubated cell surface area (7.16 × 10^-5^ m^2^ per vial, see section “Isotope Incubations for Process Rate Determination”) and the ^13^C-uptake rate per incubation vial (0.83 nmol C vial^-1^ h^-1^) resulting in 0.056 moles of inorganic carbon fixed per mole photon reaching the incubated cells. This only takes into consideration the part of the light spectrum (475–530 nm) that can be absorbed by the photopigments found in the mats (**Figure 5** and see section “Discussion”). We assumed that all cells in the incubation vials were photoautotrophic GSB, that there was no shading and that all photons hitting a cell were absorbed. Depending on absorption efficiency and the share of non-photoautotrophic cells in our incubation vials, the photoautotrophic quantum yield was likely higher than our rough estimate of 0.056 mol C (mol photon)^-1^.

### Fe-S-Biogeochemistry

**FIGURE 7:**
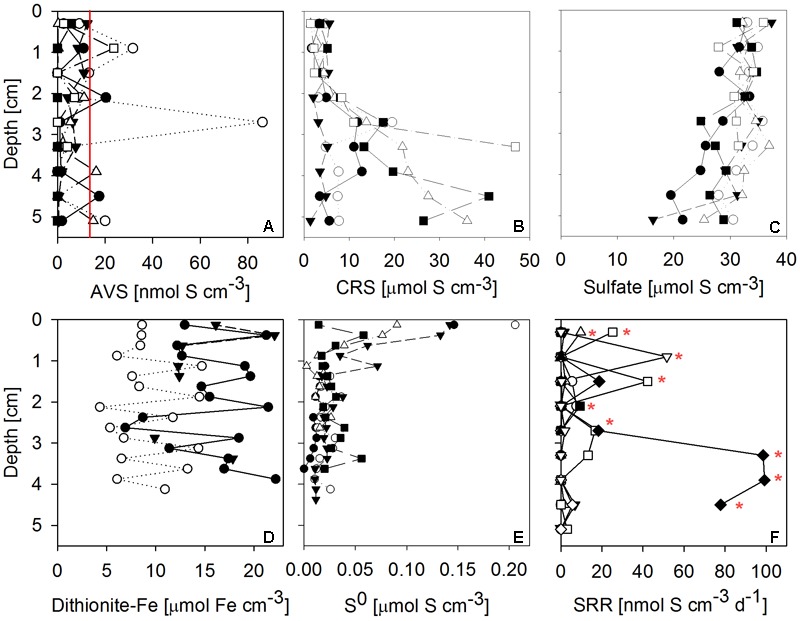
Vertical profiles of Fe-S biogeochemical parameters through microbial mat cores. Different symbols identify replicate measurements from replicate mat cores. **(A)** Solid phase AVS (= FeS + Fe_3_S_4_ + H_2_S; *n* = 6 replicate cores, the red line indicates detection limit); **(B)** solid phase CRS (= FeS_2_ + S^0^; *n* = 6); **(C)** porewater sulfate concentration (*n* = 6); **(D)** solid phase dithionite reactive iron (≈ Fe(III) + FeS; *n* = 3); **(E)** solid phase + porewater S^0^ concentrations (*n* = 5); **(F)** sulfate reduction rates (SRR; *n* = 10, red stars indicate sulfate reduction rates above detection limit). AVS, CRS and sulfate concentrations were measured on the same cores, the remaining parameters were measured on independent cores.

Sulfate reduction rates in 10 mat cores were typically very low, but highly variable between mat samples and highly heterogeneous within each core (**Figure 7F**). SRRs in the majority of subsamples fell below the detection limit. No consistent trend with depth within the mat was observed. The vertically integrated areal fluxes of sulfide caused by sulfate reduction determined in all ten samples were highly variable. Their average (30.4 nmol S cm^-2^ d^-1^) was in the range of sulfide consumption by anoxygenic photosynthesis we found in the mats. Sulfate was available in large amounts throughout the mat (**Figure 7C**). More than the other analyzed Fe-S parameters, S^0^ concentration showed a consistent vertical distribution within most mat core replicates. The highest S^0^ concentrations (average ± standard deviation: 0.12 ± 0.06 μmol S cm^-3^) were found in the top 0.25 cm decreasing to an average of 0.02 ± 0.01 μmol S cm^-3^ below 0.5 cm below the mat surface (**Figure 7E**). This indicated influx of S^0^ produced by aerobic sulfide oxidation in the halo-chemocline or *in situ* production by anoxygenic phototrophs and removal by S^0^ reduction or disproportionation below the photic layer.

Next to sulfate, dithionite reactive iron (≈ Fe(III) + FeS) and CRS (= FeS_2_ + S^0^) were by far the most abundant pools of iron and sulfur we found in the mats (**Figure 7**). High concentrations of CRS indicated that the mats were rich in pyrite given the relatively low concentrations of S^0^. FeS and H_2_S were almost absent, as indicated by low AVS values (**Figure 7A**). Since its FeS component was negligible, the abundant dithionite reactive iron largely consisted of oxidized iron [Fe(III)]. Fe(III) concentrations were in the same range as CRS concentrations. In some but not all samples, CRS showed a clear increase below 2 cm mat depth (**Figure 7B**). For dithionite reactive iron, no trend with depth could be identified.

## Discussion

### Extremely Low-Light-Adapted Anoxygenic Photosynthesis

Our results show that bacteria in the mats of MBH perform anoxygenic photosynthesis under extremely low light conditions. Based on the mat color and on the abundance of 16S rRNA genes affiliated with GSB in clone libraries, it is likely that GSB are the dominating phylum in the upper layer (**Figure 4**). The single cluster of GSB we identified in MBH mats is closely related to the extremely low-light-adapted strain *P. phaeobacteroides* BS1 (Supplementary Figure S1) from the Black Sea chemocline (Marschall et al., 2010). Pigment analysis revealed the presence of (β-)isorenieratene in all layers and BChl *e* in the upper layers of the mat, which are the typical photopigments of brown-colored GSB including *P. phaeobacteroides* BS1 (Marschall et al., 2010). The 505 nm *in vivo* absorption maximum of the brown-colored and extremely low-light-adapted *Chlorobium* strain MN1 (Overmann et al., 1992) coincided with the part of the spectrum between 475 nm and 530 nm that is relatively abundant at the cave wall (**Figure 3B**). Incubation with ^13^C-labeled compounds directly demonstrated light-dependent carbon uptake by mat microorganisms under close-to *in situ* irradiance (≤ 0.27 μmol photons m^-2^ s^-1^). Photoautotrophic fixation of DIC was considerably more efficient than photoheterotrophic incorporation of acetate (**Figure 6**). Indeed, GSB can be mixotrophic regarding their utilized carbon sources (Feng et al., 2010; Tang and Blankenship, 2010). Given that all GSB sequences in our clone libraries formed a single cluster (Supplementary Figure S1), it is likely that heterotrophic and autotrophic phototrophy were performed by the same population.

Anoxygenic photosynthesis under similarly extreme low-light conditions as observed in MBH has previously been described by phylogenetically similar bacteria in the Black Sea chemocline. Photoautotrophic activity at light intensities as low as 0.055 μmol photons m^-2^ s^-1^ *in situ* (Marschall et al., 2010) and as low as 0.015 μmol photons m^-2^ s^-1^ in laboratory cultures (Manske et al., 2005) was reported. Maximum scalar irradiance, measured on a sunny December day shortly after noon at the cave wall next to the MBH mats, was 0.084 μmol photons m^-2^ s^-1^ (**Figure 3**). Since light is strongly attenuated with depth in microbial mats (Kühl et al., 1997), cells in deeper layers will experience even less light and might display extreme phototrophic quantum efficiencies. The photoautotrophic quantum yield observed during incubation of MBH mat cells of 0.056 mol C (mol photons)^-1^ was low compared to previously reported values in GSB (Brune, 1989). However, we likely underestimated the real quantum yield in our assumption that all cells in our experimental vials were photoautotrophs which absorbed 100% of available photons of adequate wavelength. Also, sulfide and light *in situ* were much lower than under the experimental conditions these quantum yields were measured. We would expect to find a much higher quantum yield under more controlled experimental conditions.

### Light Harvesting Niches

The irradiance maxima in the UV (<400 nm) and blue-green (475–530 nm) parts of the *in situ* light spectrum (**Figure 3B**) can be understood from the absorption coefficient of water, which has its minimum between 400 and 500 nm (Sogandares and Fry, 1997). The observed shift toward short wavelengths measured at 30 m depth is thus explained by physical attenuation in the overlying water column (Sogandares and Fry, 1997), making the MBH light spectrum resemble spectra from other oligotrophic water columns (Stomp et al., 2007). Oxygenic photosynthesis in the upper water column is the most plausible explanation for the observed minimum between 400 and 475 nm in our light spectra (**Figure 3B**) as it coincides with the short-wavelength absorption peak of Chlorophylls *a*/*b* (430/460 nm). This zone is probably located above 17 m depth, where relatively rapid light attenuation with depth was observed (**Figure 2A**) and dissolved organic carbon concentration was highest.

A typical strategy of anoxygenic phototrophs from mats at shallow water depths is to harvest near-IR light of wavelengths longer than the long-wavelength maxima of chlorophylls *a*/*b* (Overmann et al., 1991). In deeper water columns including MBH, long-wavelength light is attenuated, thus phototrophic mat bacteria must make use of the remaining blue-green light (around 500 nm) and under extreme light limitation might be forced to use the UV-A (320–400 nm) parts of the spectrum. The low-light-adapted *Chlorobium* strain MN1 with the same photosynthetic pigment setup we found in MBH mats [BChl *e* and (β)-isorenieratene] has an *in vivo* absorption maximum around 500 nm (Overmann et al., 1992), which is attributable to BChl *e* (Cox et al., 1998; Glaeser et al., 2002).

Bacteriochlorophyll *e* also has an *ex vivo* absorption maximum around 340 nm within the UV-A part of the light spectrum (**Figures 5A,C**). UV light is commonly thought to be damaging rather than utilizable for phototrophic organisms due to its high energy content (Post and Larkum, 1993; Moisan and Mitchell, 2001; Xue et al., 2005). However, several studies demonstrated that UV-A light can enhance oxygenic photosynthesis (Helbling et al., 2003; Gao et al., 2007; Xu and Gao, 2010). Since irradiance <400 nm constitutes the most abundant source of light energy in their environment, anoxygenic phototrophs in MBH mats may be able to harvest UV-A light for anoxygenic photosynthesis. This hypothesis remains to be tested in future work.

### Fe-S-Biogeochemistry: The MBH Mat as Net Sulfide Sink

#### The Role of Phototrophic Sulfide Oxidation

Low sulfide (as indicated by AVS) and large amounts of pyrite-S (as indicated by high CRS and low S^0^) suggest the role of the mats as sulfide sinks (**Figure 7**). Due to the extreme light limitation, it is questionable whether phototrophic sulfide oxidation can play a quantitatively significant role in MBH sulfur biogeochemistry. Our rough estimates of sulfide fluxes from phototrophic sulfide oxidation were in the range of the mean areal SRRs (both approximately 30 nmol S cm^-2^ d^-1^), suggesting other, quantitatively more important, processes are causing the mats to be sulfide sinks.

#### The Role and Provenance of Fe-Oxides

Surprisingly large amounts of Fe-oxides were likely the main source of oxidation power in the mats. Fe(III) and CRS concentrations were in the range of values from coastal North Sea sediments (Thamdrup et al., 1994; Holmkvist et al., 2011) and sediments around Dvurechenskii mud volcano, where Lichtschlag et al. (2013) reported sulfide oxidation by Fe- or Mn-oxides. Fe-oxides can react with sulfide to form FeS and S^0^ (Jørgensen and Nelson, 2004):

(2) $$3H_{2}S+2\mathrm{FeOOH}\to S^{0}+2\mathrm{FeS}+{4H}_{2}O$$

The reaction of S^0^ with sulfide yields polysulfide (S_x_^2-^), which can enhance the rate of pyrite (FeS_2_) formation (Luther, 1991):

(3) $$\mathrm{FeS}+S_{x}{}^{2-}\to {\mathrm{FeS}}_{2}+S_{(x}{{}_{-}}_{1)}{{}^{2}}^{-}$$

A variety of reaction types for pyrite formation by reaction of sulfide with Fe(III)-minerals, Fe^2+^ and FeS are possible (discussed in Holmkvist et al., 2011). Low concentrations of AVS compared to pyrite-S in the mat suggested that the FeS quickly reacts further to form pyrite (Eq. 3), which is quite stable under reduced conditions (Fossing and Jørgensen, 1990).

The origin of very abundant Fe(III) in the mats might be dust from the Sahara, particularly the Sahel zone (Shinn et al., 2000). This Fe-containing dust was deposited episodically during climatic fluctuations (Swart et al., 2010) on the land surface, on forming soils, and in air-filled caves, which later flooded to become anchialine caves. Millimeter- to centimeter-thick red dust layers, filling crevices and niches in flooded passages, have been observed by cave divers including B. Kakuk (personal communication). Bottrell et al. (1991) found large amounts of pyrite in the cave wall of another Bahamian blue hole. Iron reducing bacteria may have settled on such iron rich dust features in the cave wall of MBH and initialized formation of the mats. Initial input of Fe(III) from the bottom of the mats in combination with continuous iron settling on the mats from the water column may explain the constant distribution of large amounts of Fe(III) throughout the mat.

Additionally, Fe(III) may be produced by iron oxidizing anoxygenic phototrophs (Widdel et al., 1993) on the mat surface. Photoferrotrophs may take up dissolved Fe^2+^ from the water column or as the product of reductive dissolution of iron from the limestone rock and also prevent the diffusive loss of Fe^2+^ produced during pyrite formation (Holmkvist et al., 2011), making the mats effective traps for dissolved and colloidal iron. This would create a niche for iron reducers, which would close the iron cycle. However, evidence for photoferrotrophy in MBH mats is lacking. *Chlorobium phaeoferrooxidans* is the first known photoferrotrophic GSB containing BChl *e*, which could theoretically enable it to harvest the part of the light spectrum available at the MBH cave wall (Llirós et al., 2015; Crowe et al., 2017). The MBH GSB cluster was neither affiliated with *C. phaeoferrooxidans* nor the other known or putative photoferrotrophic GSB *Chlorobium ferrooxidans* and *Chlorobium luteolum* (Supplementary Figure S1; Heising et al., 1999; Frigaard and Bryant, 2008).

#### Shortages of Organic Carbon and Sulfide

Despite the presence of photosynthesizing bacteria, MBH mats appear limited in chemical reductants, notably sulfide and organic carbon. Even if surplus organic carbon is produced in anoxygenic photosynthesis, it may be unavailable for organoheterotrophs, because it can be used by photoheterotrophs (**Figure 6B**). Low concentrations of nutrients and dissolved organic matter in the overlying water column were well below average lake water concentrations (Chen et al., 2015). Organic carbon shortage within the mats is suggested by low to undetectable SRRs despite high concentrations of sulfate (**Figure 7F**), which were much lower than in similar microbial mats and biofilms (Canfield and Des Marais, 1991; Kühl and Jørgensen, 1992; Visscher et al., 1992). A locally restricted anomaly of elevated SRRs in one sample may be explained by a pocket of high organic carbon, perhaps from sunken terrestrial material (**Figure 7F**).

Due to the low SRRs within the mats, the primary source of sulfide to the mats is from the water column. Vertical profiles of sulfide concentration (**Figure 2**) suggested small sources of sulfide from the bottom of the blue hole and in the halo-chemocline, where sulfate reduction has previously been observed in another Bahamian blue hole (Bottrell et al., 1991). Sulfate reduction in the halo-chemocline may be more active than in the mats due to better access to organic carbon from oxygenic phototrophy in the oxic water column, and potential anoxygenic phototrophy in the halo-chemocline (Macalady et al., 2010).

δ*-Proteobacterial* sulfate reducers and other heterotrophs seem to be abundant in the mats but their activity limited by organic carbon availability. Sulfate reduction may also experience competition for the scarce organic carbon by anoxygenic photoheterotrophy and iron reduction. While a majority of the δ*-Proteobacteria* in our clone libraries were affiliated with *Desulfobacteraceae* and *Syntrophobacteraceae* (Supplementary Figure S2), indicating that they are likely sulfate reducers (Kuever, 2014a,b), the affiliation of several other clones within the δ*-Proteobacteria* was unresolved and leaves room to speculate that they might reduce iron (Lovley et al., 2004) or disproportionate S^0^ (Thamdrup et al., 1993). Generally, the community composition of the orange and brown portions of the MBH mats was defined by groups (δ*-Proteobacteria, Chloroflexi, Planctomycetes, Aminicenantes, and Omnitrophica*; **Figure 2**) that have previously been linked to heterotrophic and fermenting carbon degradation in similar environments (Inagaki et al., 2006; Gies et al., 2014). In MBH mats, the activity of these heterotrophic bacteria must be assumed to be limited by the scarce organic carbon. We therefore propose that the mats have grown slowly but largely undisturbed over long time scales - a hypothesis that warrants future research.

### The Sulfur Cycle Was Slow in the Presence of Iron and Low Organic Carbon

Despite the presence of large amounts of sulfate, sulfur cycling within the mats was slowed down due to organic carbon shortage and the accumulations of Fe(III). Anoxygenic phototrophs were limited by light and probably also by sulfide, given slow production by sulfate reduction and competition by abiotic precipitation indicated by the pyrite accumulations. Therefore, mat-internal primary production was low and so was organic matter input from the oligotrophic water column. Low SRRs can thus be explained by organic carbon limitation.

In oxic shallow-water environments light energy can enable a phototrophic community to supply the deeper anoxic layers of a microbial mat with enough organic carbon to enable sulfate reduction to compete with FeS formation even in the presence of considerable amounts of iron (Wieland et al., 2005). If iron is present in anoxic environments under oligotrophic and light-limited conditions, sulfur cycling is slowed down considerably. In principle, this shortage in reductants would provide a niche for oxygenic photosynthesis, but the MBH light level is too low for the high energy water splitting reaction. High influx of organic carbon or sulfide would be needed to stimulate more active sulfur cycling in low-light mats.

### Conclusions and Implications

**FIGURE 8:**
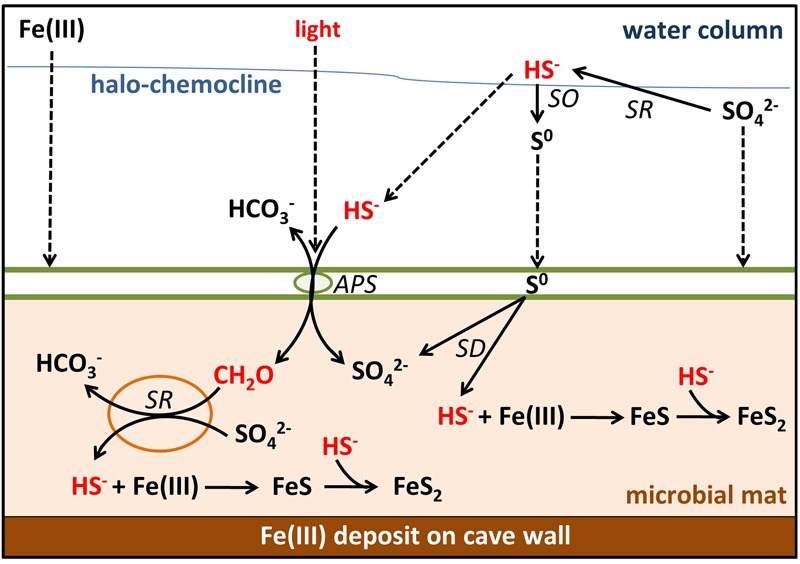
Sulfur, carbon, and iron biogeochemical cycling in the MBH microbial mat. Biotic processes are labeled SD (S^0^ disproportionation), SO (sulfide oxidation), SR (sulfate reduction), and APS (anoxygenic photosynthesis). Remaining processes are abiotic. Parameters limiting the sulfur cycle in the mat are marked with red color. Dashed arrows indicate input by sinking or diffusion from outside the mats. Green spheres = GSB; orange spheres = δ-Proteobacteria.

The biogeochemistry of MBH is characterized by limitation of energy sources like light and organic carbon. This leads to slow rates of anoxygenic photosynthesis and heterotrophic processes like sulfate reduction (**Figure 8**). Low SRRs and AVS concentrations falsified our hypothesis of the mats functioning as a net sulfide source. Instead, high concentrations of pyrite-S indicated abiotic sulfide scavenging as the mechanism responsible for making the mats a net sulfide sink. The role of very abundant δ*-Proteobacteria* in clone libraries has yet to be finally resolved, but most of them appear to be sulfate reducers whose activity is low due to organic carbon limitation.

Most sulfide produced by any *in situ* sulfate reduction or diffusing into the mat from the water column will be scavenged by abundant iron oxides rather than oxidized by light-limited anoxygenic phototrophs. Thus, little organic carbon is produced *in situ*, preventing sulfate reduction or limiting it to very low rates. In the absence of significant external input of sulfide, light and organic carbon, the sulfur cycling in these mats is slowed down. Further investigations of these mechanisms may ultimately help to understand the creation of ferruginous conditions in the presence of sulfate which is relevant for Proterozoic ocean water columns. Ferruginous conditions prevailed in the Meso- (Planavsky et al., 2011) and Neoproterozoic ocean (Canfield et al., 2008) despite the presence of approximately 1 mM sulfate (Canfield and Farquhar, 2009), which requires limitation of sulfate reduction for example by organic carbon delivery (Johnston et al., 2010; Planavsky et al., 2011).

Despite extreme light limitation, anoxygenic photosynthesis by GSB could be detected. Barely explored traits in anoxygenic photosynthesis may be hypothesized based on our data and demand further investigation including the possibility of UV-A utilization by low-light-adapted GSB. Our data show that phylogenetically very similar organisms using the same photopigment setup are responsible for anoxygenic photosynthesis in two contrasting habitats with the lowest irradiance at which photosynthesis has been reported: as planktonic cells in the Black Sea chemocline (Overmann et al., 1992) and associated as a microbial mat on the cave wall of MBH. Their photopigments BChl *e* and (β-)isorenieratene in combination with 16S rRNA from known brown-colored GSB species are therefore promising biomarkers for low-light anoxygenic photosynthesis in a range of modern and ancient environments.

## Author Contributions

SH, JM, DdB, and AF contributed to the design of the study and the experiments. VM designed and built the custom-made light sensor. SH planned and SH, BK, and JM led the field work to which TH contributed. SH did the main laboratory work, supported by AF and JK. RMR contributed a subset of the data (clone libraries and carbon concentrations) from her work in collaboration with JM and their additional exploratory data helped to guide this study. SH, DdB, JM, AF, JK, and TH contributed to data evaluation and interpretation. SH was the main author of the manuscript. SH, DdB, JM, and JK developed the intellectual content of the manuscript, to which TH and AF contributed. All authors contributed to manuscript revision and approved the submitted version.

## Conflict of Interest Statement

The authors declare that the research was conducted in the absence of any commercial or financial relationships that could be construed as a potential conflict of interest.

## Footnotes

1 http://itol.embl.de/

## Abbreviations

AVS: acid volatile sulfide
BChl: bacteriochlorophyll
CRS: chromium reducible sulfur
DIC: dissolved inorganic carbon
DOMS: diver operated microsensor system
GSB: green sulfur bacteria
MBH: magical blue hole
ORP: oxidation-reduction potential
PMT: photomultiplier
RT: retention time
