# Supplementary material for: Low-Light Anoxygenic Photosynthesis and Fe-S-Biogeochemistry in a Microbial Mat
Source: Front Microbiol. 2018 Apr 27;9:858. doi: 10.3389/fmicb.2018.00858 (PMC5934491; doi:10.3389/fmicb.2018.00858)
Supplement: Supplementary file 1 [file Data_Sheet_1.DOCX]

Supplementary Material

**Low-light Anoxygenic Photosynthesis and Fe-S-Biogeochemistry in a Microbial Mat**

Sebastian Haas^1,2*^, Dirk de Beer^1^, Judith M Klatt^1,3^, Artur Fink^1^, Rebecca McCauley Rench^4^, Trinity L Hamilton^5^, Volker Meyer^1^, Brian Kakuk^6^, Jennifer L Macalady^4^

^1^Max Planck Institute for Marine Microbiology, Bremen, Germany

^2^Department of Oceanography, Dalhousie University, Halifax, NS, Canada

^3^Department of Earth and Environmental Sciences, University of Michigan, Ann Arbor, MI, USA

^4^Geosciences Department, Pennsylvania State University, University Park, PA, USA

^5^Department of Plant and Microbial Biology, University of Minnesota, Minneapolis, MN, USA

^6^Bahamas Caves Research Foundation, Marsh Harbour, Bahamas

*** Correspondence:**Sebastian Haas
sebastian_haas@web.de; s.haas@dal.ca

## Supplementary Figures


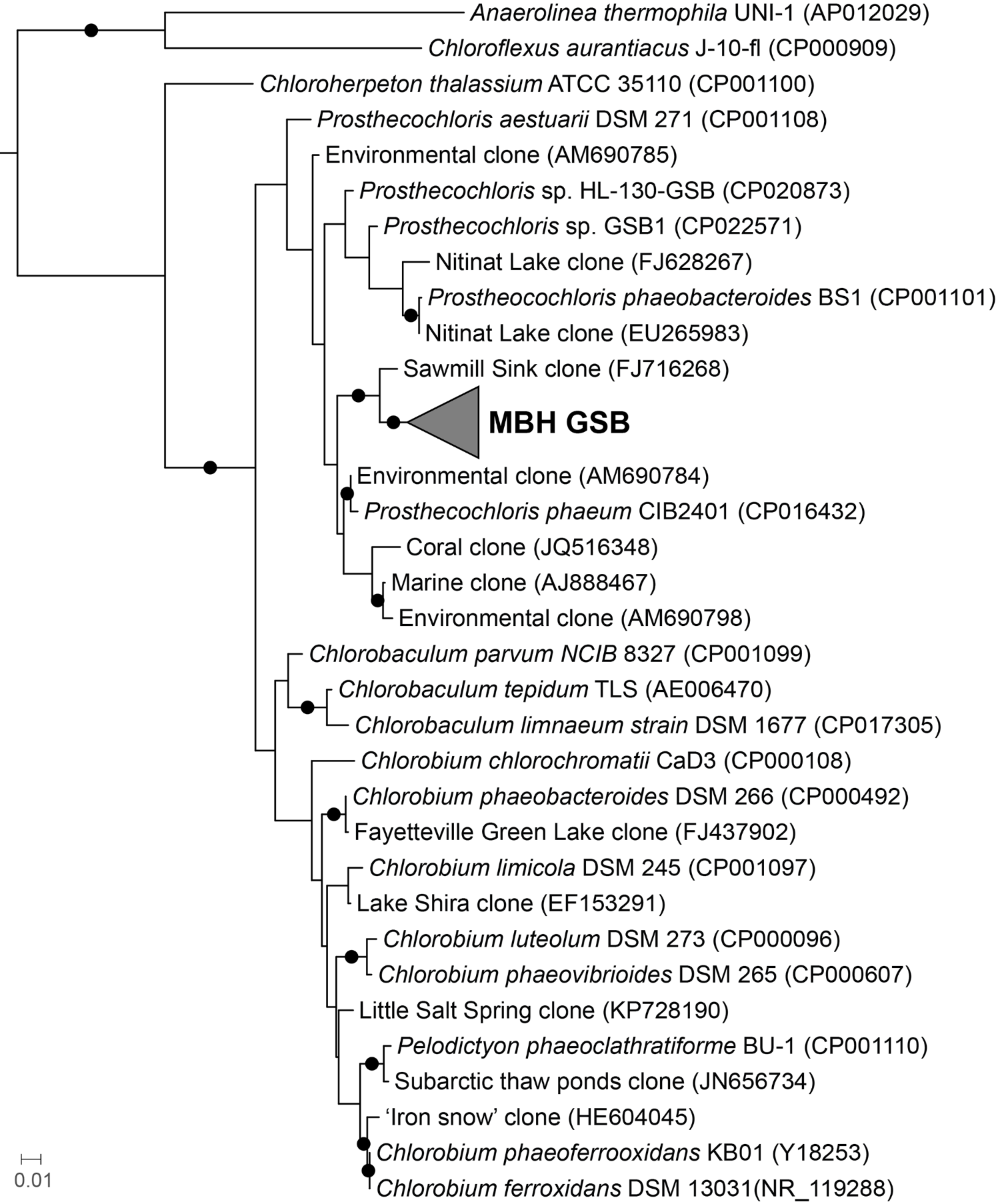


**Supplementary Figure 1.** Phylogenetic tree (maximum likelihood) of clones affiliated with green sulfur bacteria (*Chlorobi*) from the Magical Blue Hole microbial mat libraries.


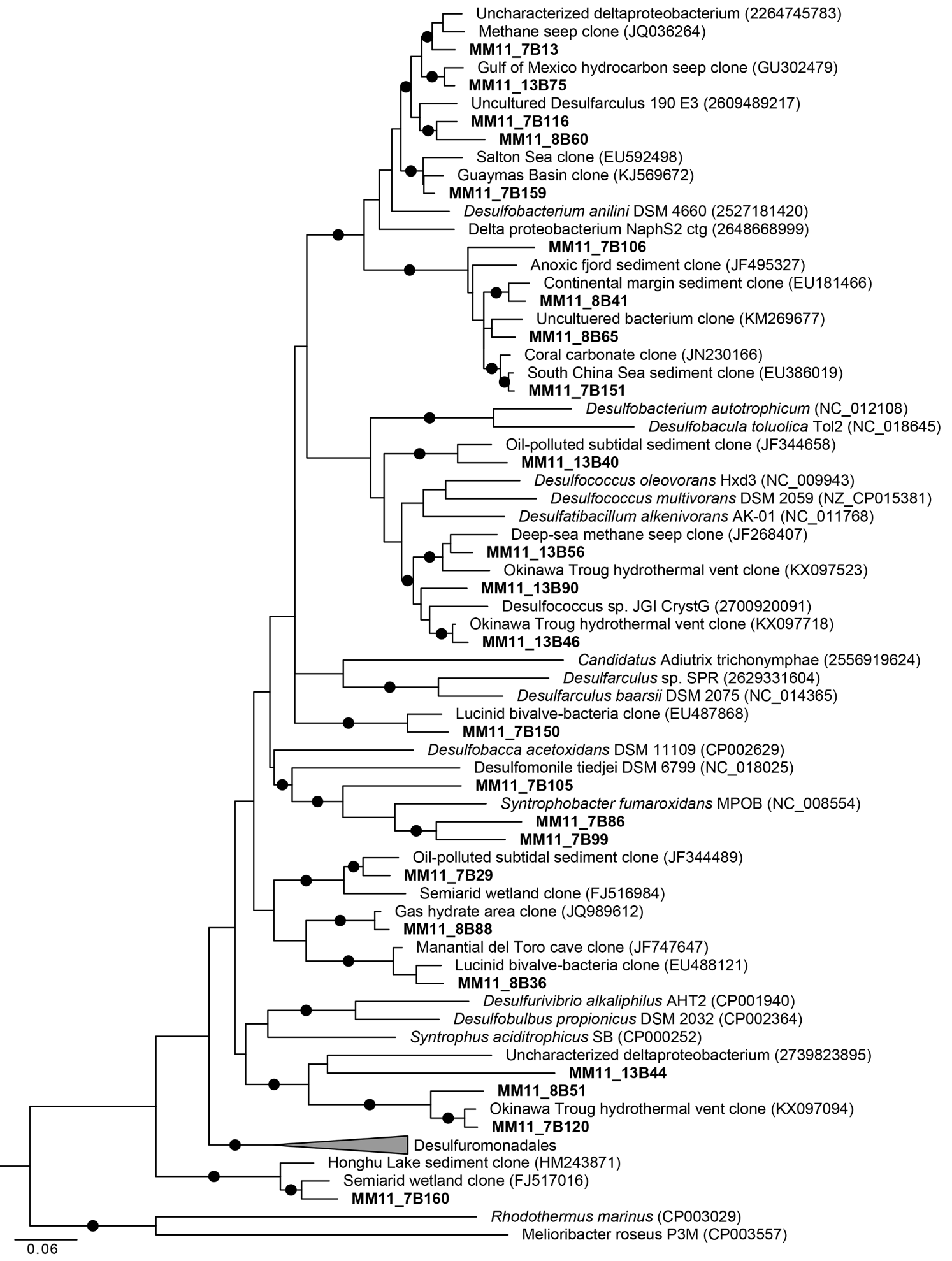
**Supplementary Figure 2.** Phylogenetic tree (maximum likelihood) of clones affiliated with *δ-Proteobacteria* from the Magical Blue Hole microbial mat libraries.
